# An exome-wide rare variant analysis of Korean men identifies three novel genes predisposing to prostate cancer

**DOI:** 10.1038/s41598-019-53445-2

**Published:** 2019-11-20

**Authors:** Jong Jin Oh, Manu Shivakumar, Jason Miller, Shefali Verma, Hakmin Lee, Sung Kyu Hong, Sang Eun Lee, Younghee Lee, Soo Ji Lee, Joohon Sung, Dokyoon Kim, Seok-Soo Byun

**Affiliations:** 1 Department of Urology, Seoul National University College of Medicine, Seoul National University Bundang Hospital, Seongnam, Korea; 2 Department of Biostatistics, Epidemiology and Informatics, Perelman School of Medicine, University of Pennsylvania, Philadelphia, PA USA; 3 Department of Genetics, Perelman School of Medicine, University of Pennsylvania, Philadelphia, PA USA; 4 Department of Biomedical Informatics, University of Utah, University of Utah School of Medicine, Salt Lake City, UT USA; 5 Complex Diseases and Genome Epidemiology Laboratory, Department of Public Health, Graduate School of Public Health, Seoul National University, Seoul, Korea; 6 Institute for Biomedical Informatics, University of Pennsylvania, Philadelphia, PA USA

**Keywords:** Prostate cancer, Genetic association study

## Abstract

Since prostate cancer is highly heritable, common variants associated with prostate cancer have been studied in various populations, including those in Korea. However, rare and low-frequency variants have a significant influence on the heritability of the disease. The contributions of rare variants to prostate cancer susceptibility have not yet been systematically evaluated in a Korean population. In this work, we present a large-scale exome-wide rare variant analysis of 7,258 individuals (985 cases with prostate cancer and 6,273 controls). In total, 19 rare variant loci spanning 7 genes contributed to an association with prostate cancer susceptibility. In addition to replicating previously known susceptibility genes (e.g., *CDYL2*, *MST1R*, *GPER1*, and *PARD3B*), 3 novel genes were identified (*FDR q* < 0.05), including the non-coding RNAs *ENTPD3-AS1*, *LOC102724438*, and protein-coding gene *SPATA3*. Additionally, 6 pathways were identified based on identified variants and genes, including estrogen signaling pathway, signaling by MST1, IL-15 production, MSP-RON signaling pathway, and IL-12 signaling and production in macrophages, which are known to be associated with prostate cancer. In summary, we report novel genes and rare variants that potentially play a role in prostate cancer susceptibility in the Korean population. These observations demonstrated a path towards one of the fundamental goals of precision medicine, which is to identify biomarkers for a subset of the population with a greater risk of disease than others.

## Introduction

Prostate cancer is a common malignancy of a gland in the male reproductive system. It is the fifth leading cancer diagnosed and the seventh leading cause of cancer deaths in Korean men^1^. The overall mortality rate in Korean men due to cancer was 188.7 per 100,000 and 6.6 per 100,000 for prostate cancer in 2014^2^. Additionally, the prevalence, incidence, and mortality of prostate cancer in Korean men have increased significantly in the past few years^1^. Heritable genetic factors contribute to the susceptibility of various cancers and the genetic attribution of the incidence of prostate cancer is more than any other cancer type^3^. Twin studies have shown that genetic factors contribute to 42% of the incidence of prostate cancer^3^. Furthermore, first degree relatives are known to have two to three-fold increased risk of developing prostate cancer^4^. These observations indicate that germline variants contribute to prostate cancer. However, the identification of germline factors involved in prostate cancer has been limited in scope, and it is still a subject of ongoing research.

In recent years, there has been a push to discover common and rare variants associated with prostate cancer among different ethnicities, as growing evidence suggests that germline factors associated with prostate cancer susceptibility may differ among different ethnicities^5^. Prostate cancer is the second leading cause of cancer mortality in American men^6^. The prevalence and mortality rates of prostate cancer differ across European, African, and Asian ethnic groups^6^. In particular, the incidence rates are particularly high in African American men and substantially low in Japanese and mainland Chinese population^6^. However, the incidence is higher among immigrant Japanese in the United States as compared Japanese living in Japan, but it is still about half that of American European population^6^. Thus, it is evident that germline factors associated with prostate cancer differ among ethnicities; therefore, in this study, we aimed to find the germline variants specific to Korean population that are associated with prostate cancer.

With the advent of high throughput genotyping technologies in the past few years, it has become easier to sequence thousands of samples. Many genome-wide association studies (GWAS) have been conducted to identify common variants associated with prostate cancer^7–9^. However, European populations are better characterized relative to Asian populations^8^. For instance, more than 100 loci have been identified in GWAS studies using European cohorts^7,8^, while studies that have performed GWAS using participants of Chinese and Japanese ethnicities have identified only 12 significant loci associated with prostate cancer^8^. Another GWAS study using common variants identified 5 significant loci associated with prostate cancer in a Korean population^9^. However, the common variants discovered to date explain only a small portion of heritability of prostate cancer, thus leaving the majority of genetic risk unexplained^10^.

Most association studies on prostate cancer to date have focused mainly on common variants. However, a proportion of the missing heritability in prostate cancer could be further explained through low-frequency and rare variants. Rare variants play a key role in the contribution to heritability among different cancer types^10,11^. Many exome-wide studies have shown rare variants associated with susceptibility genes in colorectal cancer^12^, breast cancer^13^, prostate cancer^14^, and endometrial cancer^15^. However, very few rare variant studies have been conducted in Asian populations and even fewer in the Korean population. The variants discovered using common and rare variants are rarely replicated among European, Chinese, Japanese, and Korean population studies^7–9,16^. Thus, it is important to conduct separate association studies on ethnic populations to improve our understanding of the heritability of prostate cancer among population subsets. To this end, we analyzed whole-exome array data of germline samples from Korean individuals with and without prostate cancer. Rare variants were collapsed into genes and pathways across the genome and tested for association with prostate cancer. The significant variants in the gene or pathway were further annotated and evaluated for their association with prostate cancer.

## Results

### Study design and quality control

Case population comprising 1,008 patients and a control group of 6,438 patients were obtained from the Korean Association Resource (KARE) study, which is a part of the Korean Genome and Epidemiology Study (KoGES)^17^. A summary of patient demographics for cases and controls is described in Table 1. All patients were men and of Korean ethnicity. The average ages of case and control patients were 67.43 and 54.39 years, respectively. The average body mass indices (BMI) of cases and controls were similar.

**Table 1:** Characteristics for study population showing mean, median and standard deviation of Age and Body Mass Index (BMI).

	Case (n = 1,008)	Control (n = 6,438)
**Age (years)**		
Mean	67.43	54.39
Sd	7.23	9.32
Median	68	54
**BMI (kg/m**^**2**^**)**		
Mean	24.47	24.16
Sd	8.23	3.12
Median	24.31	24.20

The quality control (QC) was performed to filter out bad samples and markers. All samples with marker call rate < 99%, sample call rate < 99%, and samples which were closely related (based on identity by descent (IBD) cutoff of 0.125) were dropped from the further analysis. After QC, 7,258 samples included 985 cases and 6,273 controls. The detailed steps involved in quality control are shown in Supplementary Figs 1 and 2. Using 71,270 variants that passed QC filters, rare variants with minor allele frequency (MAF) < 0.05 were binned into genes and association tests were performed. Further, the genomic inflation rate was calculated. A high genomic inflation rate usually indicates population substructure in data and spurious associations in the results. We found the genomic inflation factor *λ*_1000_ to be 1.196^18^ for an equivalent study of 1,000 cases and 1,000 controls (Supplementary Fig. 3). Finally, 7,258 samples with 71,270 variants that passed QC were used for subsequent statistical analysis, as described in the Methods section.

### Identification of significant genes associated with prostate cancer

**Figure 1:**
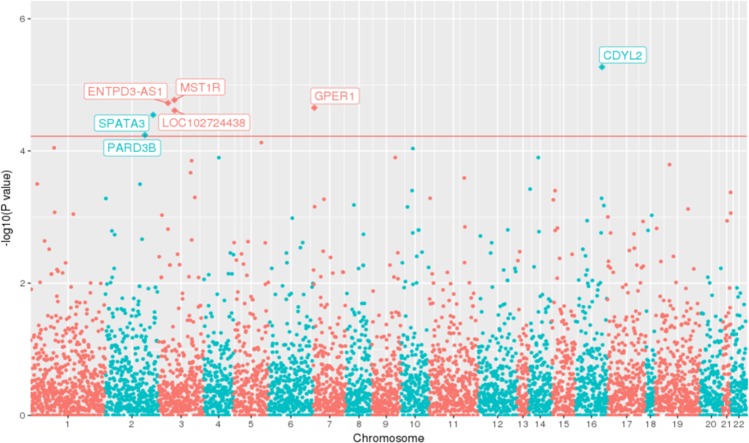
Manhattan plot showing the results of rare variant analysis. The 7 significant genes identified are shown in the Manhattan plot above the red line. The x axis highlights the chromosomes and y axis is *p-value* from the dispersion test (SKAT-O). The horizontal red line indicates genome wide significance level of *FDR q* = 0.05.

The gene-based rare variant analysis was performed to identify associations between rare variants and prostate cancer. After binning variants into 5,830 gene bins, seven genes were identified to be associated with prostate cancer (FDR < 0.05 (Fig. 1 and Table 2)). In total, 19 rare variant loci spanning across 7 genes contributed to an association with prostate cancer susceptibility (Supplementary Table 1). Three genes, including *MST1R*, *GPER1*, and *PARD3B*, have been previously implicated in prostate cancer^19–23^ and *CDYL2* in breast cancer^24^. Supplementary Fig. 4 shows regional plots for the genes with variants and exon span. Further, the distribution of MAF for the significant variants in this study population; Northeast Asians reference panel (NARD)^25^, which consists of the population from Korea (N = 850), Mongolia (N = 386), Japan, China, and Hong Kong; and gnomeAD^26^ are shown in Supplementary Table 2. The samples that had more than one rare variant among significant locus are shown in Supplementary Fig. 5.

**Table 2:** Gene-based rare variant analysis results using SKAT-O.

Gene	Chr: Build 38 position	# variants	cMAF	cMAF Case	cMAF Control	SKAT-O p-value	FDR q-value
CDYL2	16: 80597899-80805043	2	0.00055	0.00050	0.00056	5.39E-06	0.0277
MST1R	3: 49886471-49903637	5	0.01707	0.02066	0.01651	1.70E-05	0.0277
ENTPD3-AS1	3: 40390951-40453308	2	0.00034	0.00101	0.00024	1.89E-05	0.0277
GPER1	7: 1086807-1093815	2	0.02891	0.03175	0.02846	2.23E-05	0.0277
LOC102724438	3: 49899191-49925038	2	0.00296	0.00554	0.00255	2.45E-05	0.0277
SPATA3	2: 230996124-231019939	2	0.00372	0.00706	0.00319	2.85E-05	0.0277
PARD3B	2: 204545793-205620162	6	0.04163	0.04234	0.04154	5.76E-05	0.0480

Chr: Chromosome; cMAF: cumulative minor allele frequency of all variants included in the gene/bin;

Seven genes significantly associated with prostate cancer after adjusting the SKAT-O *p-value* for multiple tests.

Further, the rare association tests were rerun by removing one variant at a time from the gene/bin to elucidate the significance of that particular variant in the gene/bin (Supplementary Table 1). A decrease in the significance of the gene (increase in p-value) represents a significant contribution of the variant while maintaining a significant association suggests that the contribution of the variant is insignificant. In case of *MST1R* and *LOC102724438*, when the stop gained variant, rs200626206 was removed and the gene was insignificant (*p*_*rm*_ > 0.05)_,_ indicating that it is the most significant variant in the gene/bin, as most of the signal detected in the genes is attributed to rs200626206 loci (Supplementary Table 1). Nonsense mutations often produce nonfunctional protein products due to premature termination of translation, thus the significant contribution of rs200626206 loci to the signal was expected. The variants rs181756759 and rs201829385 in genes *GPER1* and *PARD3B*, respectively, have insignificant *p*_rm_ and are predicted to be ‘probably damaging’ with Polyphen score > 0.95 (Supplementary Table 1). Thus, there is a high possibility that these variants result in partial or complete loss of protein function^27,28^.

### Variant annotation

To characterize the clinical significance, effect of variants on the protein, and implications in human inherited diseases, the variants within significantly associated genes were annotated using ClinVar and Variant Effect Predictor (VEP). None of the significant variants binned were found in ClinVar. VEP annotated the variants concerning their effect on the coding region (Supplementary Table 1). VEP also annotates variants by their potential influence on protein sequence (e.g., high, moderate, and low). High impact variants have a disruptive effect on proteins, such as protein truncation and loss of function. Moderate effect variants are non-disruptive but can change protein sequence while low effect variants are unlikely to change protein behavior.

Here, VEP categorized 16 variants as a moderate effect, 2 variants as high effect, and 1 variant as a modifier (Supplementary Table 1). Therefore, most rare variants within the coding regions of genes associated with prostate cancer possibly influence protein function.

### Identification of significant pathways associated with prostate cancer

Pathway analysis was performed using variants and genes discovered by gene-based rare variant association test. The biological pathways were derived using Ingenuity Pathway Analysis (IPA, QIAGEN Redwood City, www.qiagen.com/ingenuity), and 4 canonical pathways were identified, including IL-15 Production, MSP-RON Signaling Pathway, Sperm Motility and IL-12 Signaling, and Production in Macrophages (Table 3). IL-15 Production, MSP-RON Signaling. Further, pathway enrichment test was also run using EnrichR, which identified estrogen signaling pathway, signaling by MST1 pathway, and four Gene Ontology (GO) molecular function ontologies^29,30^.

**Table 3:** Pathways/Ontologies discovered by Ingenuity pathway analysis, KEGG, Reactome, WikiPathways and GO molecular function ontology.

Database	Pathway/Ontologies	p-value
WikiPathways (2019)	Estrogen signaling pathway WP712	0.008023
KEGG (2019)	Estrogen signaling pathway	0.04698
Reactome (2016)	Signaling by MST1_Homo sapiens_R-HSA-8852405	0.001749
Ingenuity canonical pathway	IL-15 Production	0.004
	MSP-RON Signaling Pathway	0.01
	Sperm Motility	0.018
	IL-12 Signaling and Production in Macrophages	0.022
GO molecular function (2018)	MAP kinase activity (GO:0004709)	0.02733
	Transmembrane receptor protein tyrosine kinase activity (GO:0004714)	0.02150
	Transmembrane receptor protein kinase activity (GO:0019199)	0.02185
	Mitogen-activated protein kinase kinase binding (GO:0031434)	0.02288
	Phosphatidylinositol binding (GO:0035091)	0.03482

The pathways discovered by Ingenuity pathway analysis using the 19 rare variants present in the 7 significant genes associated with prostate cancer.

The WikiPathways, KEGG, Reactome, GO molecular function were generated using gene set enrichment analysis web server Enrichr^29,30^.

## Discussion

In this work, an exome-wide rare variant analysis study was performed, and 19 novel low-frequency variants in 7 genes were identified to be associated with prostate cancer in Korean men. Since common variants alone do not completely explain the heritability of prostate cancer, integrative analyses of rare variants across the genome can provide us with a new understanding of prostate cancer heritability. Though common variants have been studied previously in the Korean population, this is the first exome-wide study of rare variants associated with prostate cancer in the Korean population. The genes and variants discovered in this study can potentially help in early diagnostic and understanding of carcinogenesis in prostate cancer.

The rare variants were binned into genes and association tests were performed across the genes. Binning of rare variants increases statistical power to detect rare variant associations^31^ and helps interpret the effect of rare variants on the prognosis and progression of prostate cancer. Several genes identified in this study were previously implicated with prostate cancer and other cancers. *In-vivo* studies have shown an association of *MST1R*, *GPER1*, and *PARD3B* with prostate cancer^19–23^. Another gene we found, *CDYL2*, has common variants that are implicated in breast cancer^24^. Since three of the genes discovered in this study have been previously validated to be associated with prostate cancer, it would appear that our analysis predicted true associations.

Angiogenesis, cell survival, and cell proliferation are hallmarks of cancer^32^. One of the genes that we found was significantly associated with prostate cancer, *MST1R* (Macrophage Stimulating 1 Receptor) or *RON*, is overexpressed in prostate cancer and various other cancers^33^. *RON* is known to be overexpressed in breast cancer and bladder cancer and is associated with poor clinical outcome^33^. *In vivo* study using genetically engineered mouse model has shown that the *RON* receptor plays a functional role in prostate tumor and that deficient *Ron* receptor signaling is associated with smaller tumor size^34^. Another study on stromal cells of the prostate tumor using mouse model showed that loss of *Ron* in tumor-associated macrophages inhibits cancer cell growth^19^. Another gene significantly associated with prostate cancer, *GPER1* (G Protein-Coupled Estrogen Receptor 1) or *GPR30*, is known to regulate cell growth by non-genomic signaling of estrogen^35^. *GPER1* is also known to stimulate cell proliferation in breast, endometrial, ovarian, and thyroid cancer cells by rapid but transient activation of Erk1/2^35^. Besides, in the case of prostate cancer, *GPER1* is known to control cancer cell growth through *GPER1* mediated pathways^35^. The presence of alternate allele in rs11544331, one of the rare variant loci binned in *GPER1*, is known to result in the expression of P16L variant of *GPER1*^36^. The substitution of proline with leucine at position 16 of the *GPER* protein sequence blocks *GPER* from being glycosylated and causes it to localize to the nucleus, although typically it should localize outside of nucleus^36^. The *P16L* in the nucleus may also regulate transcription of cancer-relevant genes and migration of carcinoma cells^36^.

Of the genes identified in this study, *PARD3B* (Partitioning defective 3 homolog) plays an essential role in asymmetric cell division, polarized growth, and maintenance of cell-polarity^37^. Mutational inactivation of its homolog gene *PARD3* is known to cause carcinogenesis in prostate cancer^38^. *In vivo* studies have shown that downregulation of Par3 in breast cancer induces cell invasion and metastasis by decreasing cell-cell cohesion in a Tiam1/Rac-GTP pathway-dependent manner^23^. Higher expression of *PARD3B* is associated with colorectal cancer malignancy and poor survival, as *PARD3B* inhibits Lkb1/AMPK signaling pathway and its knockout induces apoptosis and reduces proliferation, supporting its role in colorectal cancer cell survival^39,40^. Additionally, a previous genome-wide association study found rs2335704, which resides in *PARD3B*, to be associated with tuberculosis^41^. Another gene, *CDYL2* (Chromodomain Y Like 2 or Prostate Cancer Candidate Protein 1), is involved in catalytic activity, protein binding, and methylated histone binding^42^. Genome-wide studies using common variants have identified loci in *CDYL2* associated with breast cancer^43^.

*ENTPD3-AS1* is a long non-coding RNA that we found was significantly associated with prostate cancer. A locus (rs193921050) in *ENTPD3-AS1* has been reported for ‘Malignant tumor of prostate’ in ClinVar with uncertain clinical significance and review status of 0/4^44^. The mutation on the locus was discovered in somatic tissue but was not found to be mutated at a significantly higher rate relative to the background mutation rate^45^.

In addition to the genes, the estrogen signaling pathway was significantly enriched using WikiPathways and KEGG pathway. Evidence suggests that prostate carcinogenesis and progression involves local estrogen signaling mechanisms^46,47^. Further, signaling by MST1 pathway was enriched using the Reactome pathway database. An *in vitro* study showed that MST1 suppressed prostate cancer growth^48^. Moreover, MST1 is the key kinase component of the Hippo-YAP pathway, which restricts prostate cancer progression by interacting with multiple molecular pathways^49^. The Ingenuity pathway analysis revealed three significant pathways. IL-15 production pathway, one of the significant pathways, is known to be associated with prostate cancer. The expression of IL-15 is known to decrease the migration, invasion, and angiogenesis but increase tumor volume by increasing lipid deposition and inflammation in prostate cancer^50^. IL-15 also alters the expression of genes involved in cell death and immune response^50^. Vaccinations using IL-15 are effective in up-regulating immune responses, reducing invasion, and improving survival^51^. Another pathway, MSP-RON signaling pathway, has been previously known to be associated with many cancer types, including prostate cancer, and has been extensively studied *in vivo* and *in vitro*. The MSP-RON signaling generates oncogenic variants and activates downstream pathways, resulting in tumorigenesis, proliferation, angiogenesis, invasion, and resistance to chemotherapy^52^. Loss of RON in myeloid cells has been shown to reduce prostate cancer growth in mice models^52^. IL-12 signaling is anti-carcinogenic, and IL-12 deficiency in mice is known to induce the development of spontaneous tumors and promote their growth^53^. Further, the GO molecular function ontology enrichment indicated four significantly enriched molecular function ontologies, including Mitogen-activated protein kinase (MAPK) activity, Transmembrane receptor protein tyrosine activity, Transmembrane receptor protein kinase activity, and Phosphatidylinositol binding. All the ontologies discovered are known to be associated with prostate cancer^33,54,55^.

Even though some of the genes we found have already been implicated, we found 19 novel variants and 3 novel genes that are associated with prostate cancer. Moreover, all the pathways that were found to be associated have been well studied and have been found to play a key role in cancer. All the variants discovered were missense and stop gained, except one variant in an intron, as categorized by VEP. Variation in amino acid sequence could potentially affect stability, conformational dynamics, drug response, and other protein properties that could result in a pathological condition and increased susceptibility to disease^56^. Many variants were also filtered out when re-clustering and filtering were performed using CHARGE criteria, a more relaxed approach shown in Park *et al*., which could be used to preserve more variants while maintaining the genotyping accuracy of common and rare variants^57^. Further studies would be required to validate our findings, as variants discovered in this study were not discovered in the European population studies^58^. This is probably due to different genetic factors affecting prostate cancer susceptibility among different ethnic groups^5^. Additional exploration of the molecular mechanisms is required to understand the exact role of the variants in prostate cancer. Further studies are also required to elucidate the role of lifestyle/environment, especially dietary factors, in the Korean population, as they have been previously shown to be associated with prostate cancer^4^. In conclusion, we found novel genes and rare variants that are associated with prostate cancer in the Korean population, revealing potential biomarkers for prostate cancer that are unique to Korean ethnicity. They could also help us explain the missing heritability in prostate cancer, which could be applied in the field of precision medicine.

## Methods

### Samples and data set

Between November 2003 and July 2013, we prospectively recruited 1,008 prostate cancer patients from a single tertiary hospital, Seoul National University Bundang Hospital, and conducted a case-control study that included 6,438 age-matched controls from the Korean Association Resource (KARE) study, which was a part of the Korean Genome and Epidemiology Study (KoGES)^17^. Blood specimens were prospectively collected throughout the course of the study from all of the prostate cancer patients. The automatic firing mechanism was used to perform transrectal ultrasound-guided multi-core (≥12) biopsies bilaterally near the base, mid-gland, and apex, with at least six biopsies per side. A total of 12 baseline biopsy cores were taken from all of the men, and additional biopsies of suspicious lesions were obtained if needed. Further, 820 patients among the study population who had prostate cancer were treated with radical prostatectomy (RP) in the same hospital. The genotyping was done using the blood samples collected.

### Exome chip

The Illumina HumanExome BeadChip 12v1-1 system provides 242,901 variants selected over 12,000 individual human exome and whole-genome sequences representing diverse populations and ethnicities. The chip focuses on protein-altering variants. A more detailed explanation is available at http://genome.sph.umich.edu/wiki/Exome_Chip_Design.

### Genotyping and quality control

The datasets were generated using Illumina HumanExome BeadChip 12v1-1. We used Illumina’s GenTrain version 2.0 clustering algorithm with the GenomeStudio software (V2011.1) for genotype calling. The genotype calling for the exome chip was performed following the best practices defined in Grove *et al*.^59^. We performed manual re-clustering and visual inspection using CHARGE clustering method^59^ to improve the accuracy of variant calling (Supplementary Fig. 6). A separate study on the quality of variants showed that re-clustering using CHARGE criteria on KoGES dataset with more relaxed cutoffs has 99.9% concordance rate for rare variants with whole-exome sequencing data, which indicates that rare variant calls are robust^57^. Quality control filters were applied to both case and control datasets separately. Since the number of variants differed in the datasets, only common variants between cases and controls were selected for the analysis. The datasets were merged after QC, and filters were again applied to the merged dataset. As a part of the quality control, sample call rates, marker call rates, and sample relatedness were checked^60^. The palindromic SNPs and SNPs with indels were removed. The samples and markers with a call rate of less than 99% were removed. Identity by descent (IBD) was calculated using plink, and IBD threshold of 0.125 was used to remove related samples. The detailed quality control steps are shown in Supplementary Figs 1 and 2.

The final merged dataset had 7,258 samples with 985 cases and 6,273 controls and 71,270 variants. The dataset was checked for batch effects. Since datasets from different sources were merged, population stratification in the data could have occurred^60^. Principal Component Analysis was performed using SMARTPCA^61^ on the dataset after the LD pruning using plink option ‘-indep-pairwise 50 5 0.2’ and removing all SNPs with MAF < 0.05. PCA was performed to check case and control sample clusters (Supplementary Fig. 7). PCA was also performed by projecting onto 1,000 genomes data. The case and controls clustered together around the South Asian population, as shown in Supplementary Fig. 8. Further, quantile-quantile (Q-Q) plot was drawn using SKAT-O p-values to check for inflation (Supplementary Fig. 3).

### Rare variant gene-based association test

The rare variant analysis was performed using BioBin (https://ritchielab.psu.edu/software/biobindownload), a tool that can be used to perform rare variant burden tests^31^. BioBin bins all variants into gene bins and variants outside genes into intergenic region bins. Subsequently, SKAT-O was used to test for statistical significance of associations^62^. SKAT-O increases statistical power by optimally combining burden and dispersion (SKAT) tests and adaptively applying them^62^. Since rare variants are statistically underpowered for the association test, binning of rare variants by biologically informed units, such as gene or pathway, increases statistical power to detect rare variant associations by increasing the composite allele frequency and reducing the degrees of freedom^31^.

BioBin is configured by default to bin all variants with minor allele frequency (MAF) below 5%. Library of Knowledge Integration (LOKI) is a database of genomic locations of SNPs and genes as well as known relationships among genes and proteins, such as interaction pairs, pathways, and ontological categories integrated from various disparate data sources^31^. LOKI provides prior knowledge to BioBin^31^. All variants with MAF < 0.05% were removed. Variants with MAF > 0.05% in case population or control population were included, and only genes with at least 2 variants were tested. Age and first 5 principal components were incorporated as covariates to adjust for age and population stratification. The first 5 principal components that defined maximum variance were selected, as shown in Supplementary Fig. 9. The weight-loci argument was used to add Madsen & Browning weights to each locus^63^. BioBin creates bin based on gene regions when the bin-regions argument is set using gene information from LOKI. The false discovery rate (FDR) correction was applied to adjust for multiple testing. Any FDR adjusted *q-value* < 0.05 was considered significant. The rare association tests were run again by removing one variant at a time from the significant bins to ascertain the significance of the variant. The higher the SKAT-O *p-value* (*p*_rm_), the more significant is the contribution of the variant in the bin.

### Variant annotation

ClinVar is a public archive that connects human variation to phenotypes, clinical significance, relationship to human health, and other supporting data obtained through submissions by various groups^44^. These are aggregated to reflect both consensus and conflicting assertions^44^. Variant effect predictor (VEP) provides information about the variants’ location, gene/transcript affected by variants, types of mutation (i.e., stop gained, missense, stop-lost, and frameshift), and protein change scores, which indicate possible partial/complete loss of function of the protein due to amino acid substitution. All the variants in significantly associated genes were annotated using ClinVar and VEP.

### Pathway enrichment analysis

The significant variants and genes were used for pathway enrichment analysis using Ingenuity pathway analysis and Enrichr. Enrichr is a web-based enrichment analysis tool which contains pathway and ontology libraries from various sources^29,30^. The WikiPathways (2019), KEGG (2019), Reactome (2016), and GO molecular function (2018) libraries were used as part of Enrichr.

### Ethics statement

This study was approved by our institutional review board (Seoul National University Bundang Hospital Institutional review board; IRB number, B-1312/232-302) and followed the rules stated in the Declaration of Helsinki. All participants provided written informed consent.

## Supplementary information

Supplementary info
