# Author Correction: An exome-wide rare variant analysis of Korean men identifies three novel genes predisposing to prostate cancer

**DOI:** 10.1038/s41598-020-59361-0

**Published:** 2020-02-07

**Authors:** Jong Jin Oh, Manu Shivakumar, Jason Miller, Shefali Verma, Hakmin Lee, Sung Kyu Hong, Sang Eun Lee, Younghee Lee, Soo Ji Lee, Joohon Sung, Dokyoon Kim, Seok-Soo Byun

**Affiliations:** 1 Department of Urology, Seoul National University College of Medicine, Seoul National University Bundang Hospital, Seongnam, Korea; 2 Department of Biostatistics, Epidemiology and Informatics, Perelman School of Medicine, University of Pennsylvania, Philadelphia, PA USA; 3 Department of Genetics, Perelman School of Medicine, University of Pennsylvania, Philadelphia, PA USA; 4 Department of Biomedical Informatics, University of Utah, University of Utah School of Medicine, Salt Lake City, UT USA; 5 Complex Diseases and Genome Epidemiology Laboratory, Department of Public Health, Graduate School of Public Health, Seoul National University, Seoul, Korea; 6 Institute for Biomedical Informatics, University of Pennsylvania, Philadelphia, PA USA

Correction to: *Scientific Reports* 10.1038/s41598-019-53445-2, published online 20 November 2019

In the original version of this Article, Soo Ji Lee was incorrectly affiliated with ‘Department of Biomedical Informatics, University of Utah, University of Utah School of Medicine, Salt Lake City, UT, USA’. The correct affiliation is listed below.

Complex Diseases and Genome Epidemiology Laboratory, Department of Public Health, Graduate School of Public Health, Seoul National University, Seoul, Korea

Additionally, the Author Contributions section in this Article was incomplete.

“J.J.O., M.S., D.K. and S.S.B. conceived the project. J.J.O., H.L., S.K.H., S.E.L. and S.S.B. curated data. J.J.O., M.S., J.M., S.V., Y.L., S.J.L., J.S. performed the formal analysis along with visualization. D.K. and S.S.B. provided resources. J.J.O., M.S., J.M., D.K. and S.S.B. supervised all aspects of this work. The original draft was written by J.J.O. and M.S. All authors reviewed and edited the manuscript.”

now reads:

“J.J.O., M.S., D.K. and S.S.B. conceived the project. J.J.O., H.L., S.K.H., S.E.L. and S.S.B. curated data. J.J.O., M.S., J.M., S.V., Y.L., S.J.L., J.S. performed the formal analysis along with visualization. D.K. and S.S.B. provided resources. J.J.O., M.S., J.M., D.K. and S.S.B. supervised all aspects of this work. The original draft was written by J.J.O. and M.S. All authors reviewed and edited the manuscript. Jong Jin Oh and Manu Shivakumar contributed equally.”

These errors have now been corrected in the PDF and HTML versions of the Article and in the Supplementary Information file which accompanies the Article.

