# Supplementary material for: An exome-wide rare variant analysis of Korean men identifies three novel genes predisposing to prostate cancer
Source: Sci Rep. 2019 Nov 20;9:17173. doi: 10.1038/s41598-019-53445-2 (PMC6868235; doi:10.1038/s41598-019-53445-2)
Supplement: Supplementary file 1 — Supplementary info [file 41598_2019_53445_MOESM1_ESM.docx]

**Supplementary Tables and Figures**

**An exome-wide rare variant analysis of Korean men identifies three novel genes predisposing to prostate cancer**

Jong Jin Oh^1§^, Manu Shivakumar^2§^, Jason Miller^3^, Shefali Verma^3^, Hakmin Lee^1^, Sung Kyu Hong^1^, Sang Eun Lee^1^, Younghee Lee^4^, Soo Ji Lee^4^, Joohon Sung^5^,

Dokyoon Kim^2,6*^, Seok-Soo Byun^1*^

^1^Department of Urology, Seoul National University College of Medicine, Seoul National University Bundang Hospital, Seongnam, Korea

^2^Department of Biostatistics, Epidemiology and Informatics, Perelman School of Medicine, University of Pennsylvania, Philadelphia, PA, USA

^3^Department of Genetics, Perelman School of Medicine, University of Pennsylvania,

Philadelphia, PA, USA

^4^Department of Biomedical Informatics, University of Utah, University of Utah School of Medicine, Salt Lake City, UT, USA

^5^Complex Diseases and Genome Epidemiology Laboratory, Department of Public Health, Graduate School of Public Health, Seoul National University, Seoul, Korea

^6^Institute for Biomedical Informatics, University of Pennsylvania, Philadelphia, PA, USA

*Author for correspondence

**Key words:** Prostate cancer, germline variants, rare variant analysis, Korean population, Health disparity

Conflict of interest statement: The authors declare no conflicts of interest.


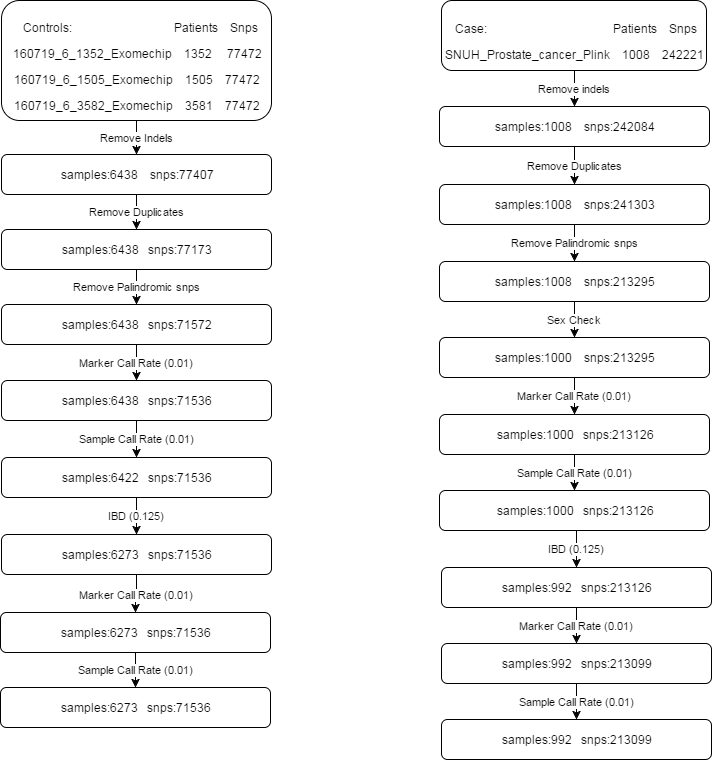


**Supplementary Figure 1.** Flowcharts of different initial quality control measures applied to the control and case data separately. In the initial step control data is merged, followed by removal of indels, duplicates and palindromic SNPs. All the samples with marker call rate below 99% and markers with samples below 99% are removed. Closely related samples (IBD = 0.125) are removed. Data is again filtered for sample call rate and marker call rates.


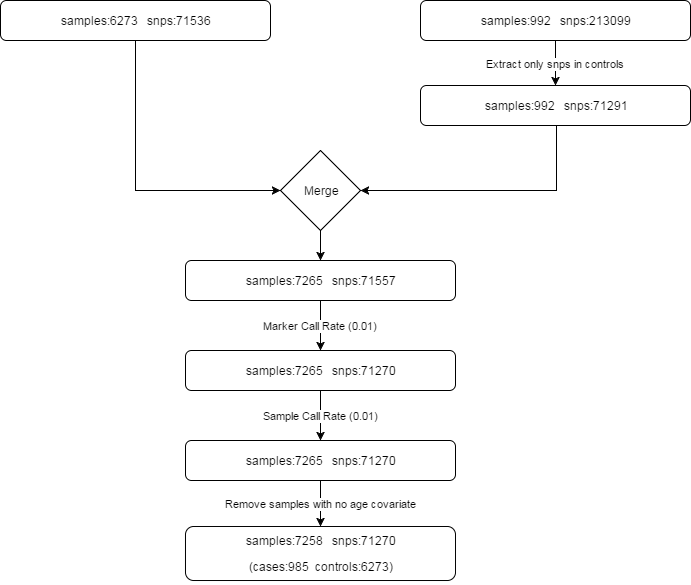


**Supplementary Figure 2.** Merging case and control dataset and quality control steps applied to the merged dataset. All the samples with marker call rate below 99% and markers with samples below 99% are removed. Samples with missing age are removed to get 985 cases and 6273 controls.


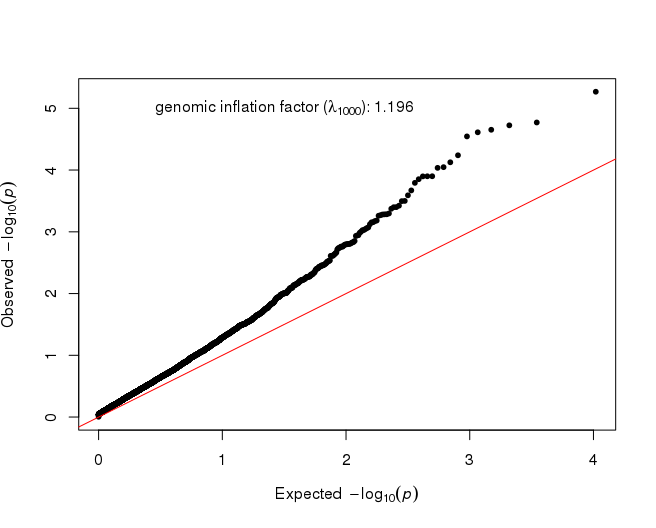


**Supplementary Figure 3.** QQ plot of SKAT-O *p-values* from rare variant analysis. The genomic inflation rate *λ*_1000_ was 1.196.


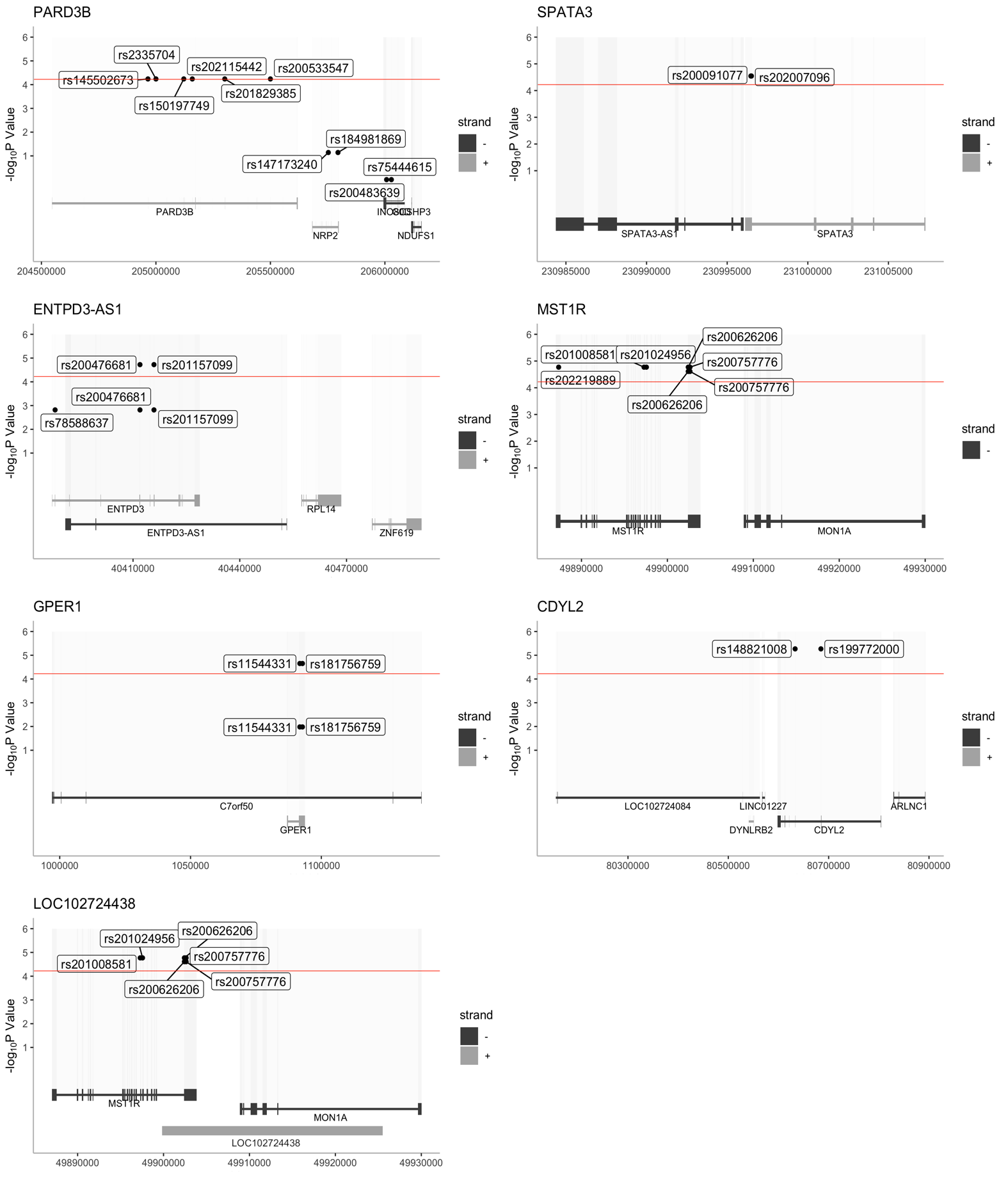


**Supplementary Figure 4.** Regional plots showing all significant variants and genes. The x axis represents the chromosome, build 38 start and stop positions and y axis is -log transformed *p-values* from burden test (SKAT-O). The horizontal red line represents the -log transformed p-value at FDR=0.05.


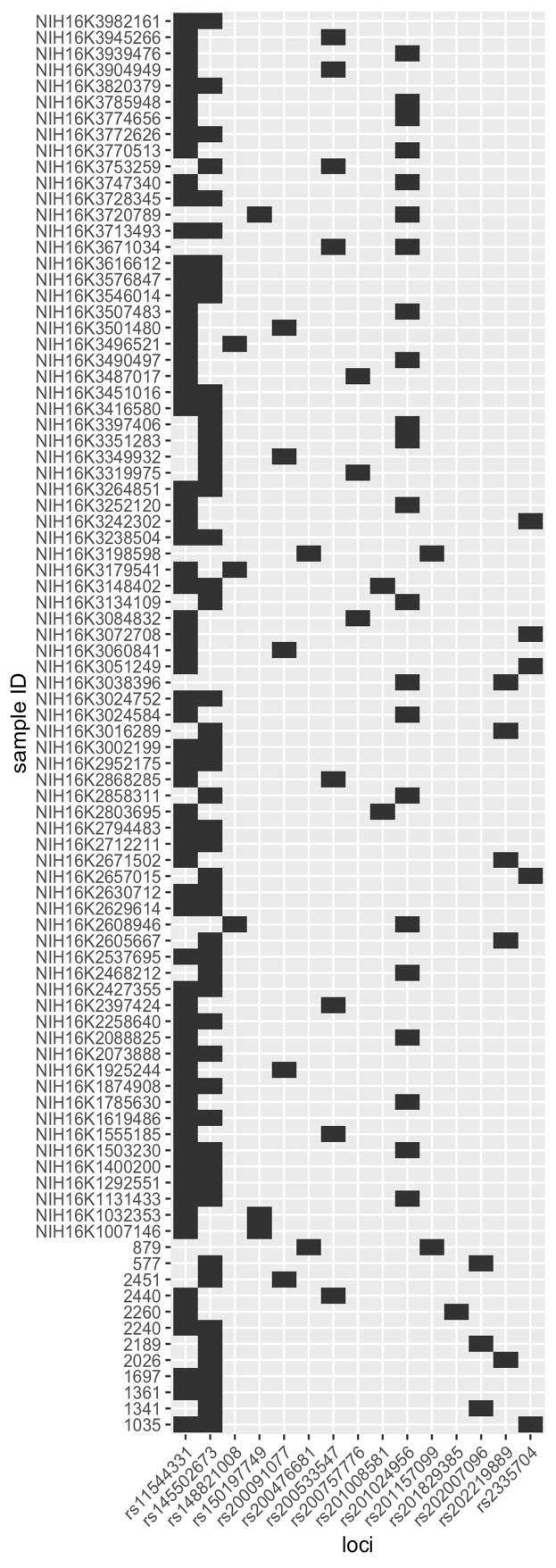


**Supplementary Figure 5.** Samples with rare variants in more than one significant locus.


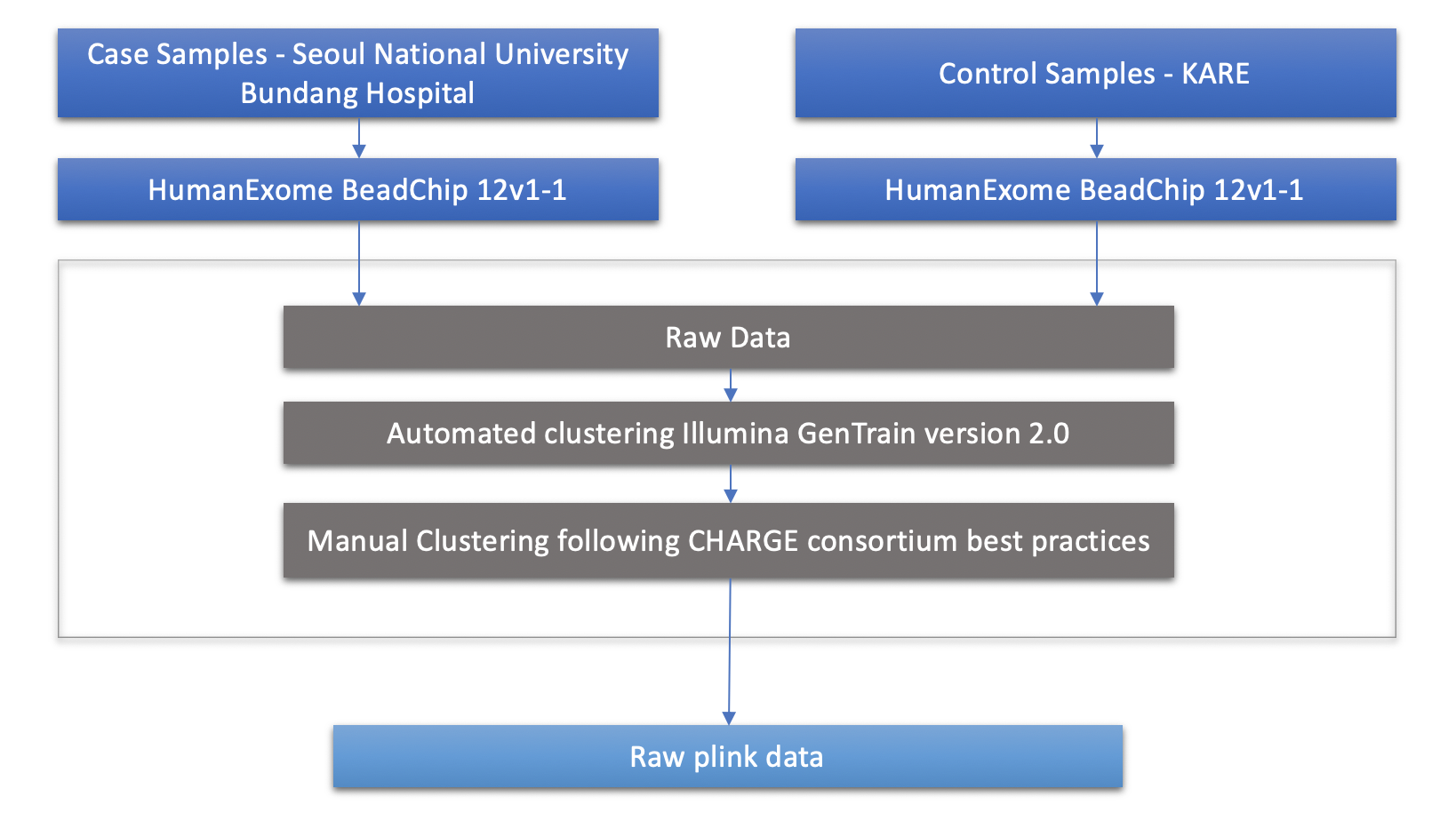


**Supplementary Figure 6.** Workflow of genotype calling. The genotype calling on cases and controls were done separately.


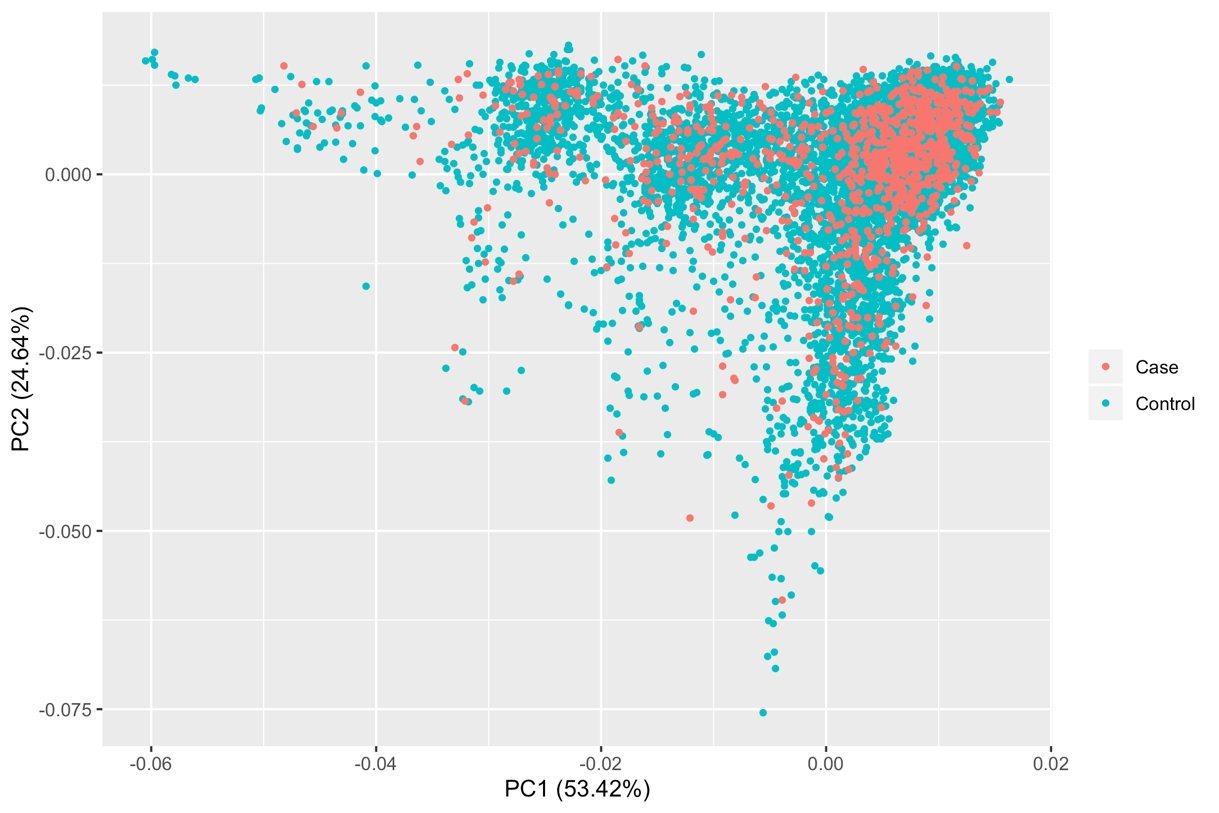


**Supplementary Figure 7.** Principle Component Analysis (PCA) Case and Controls.


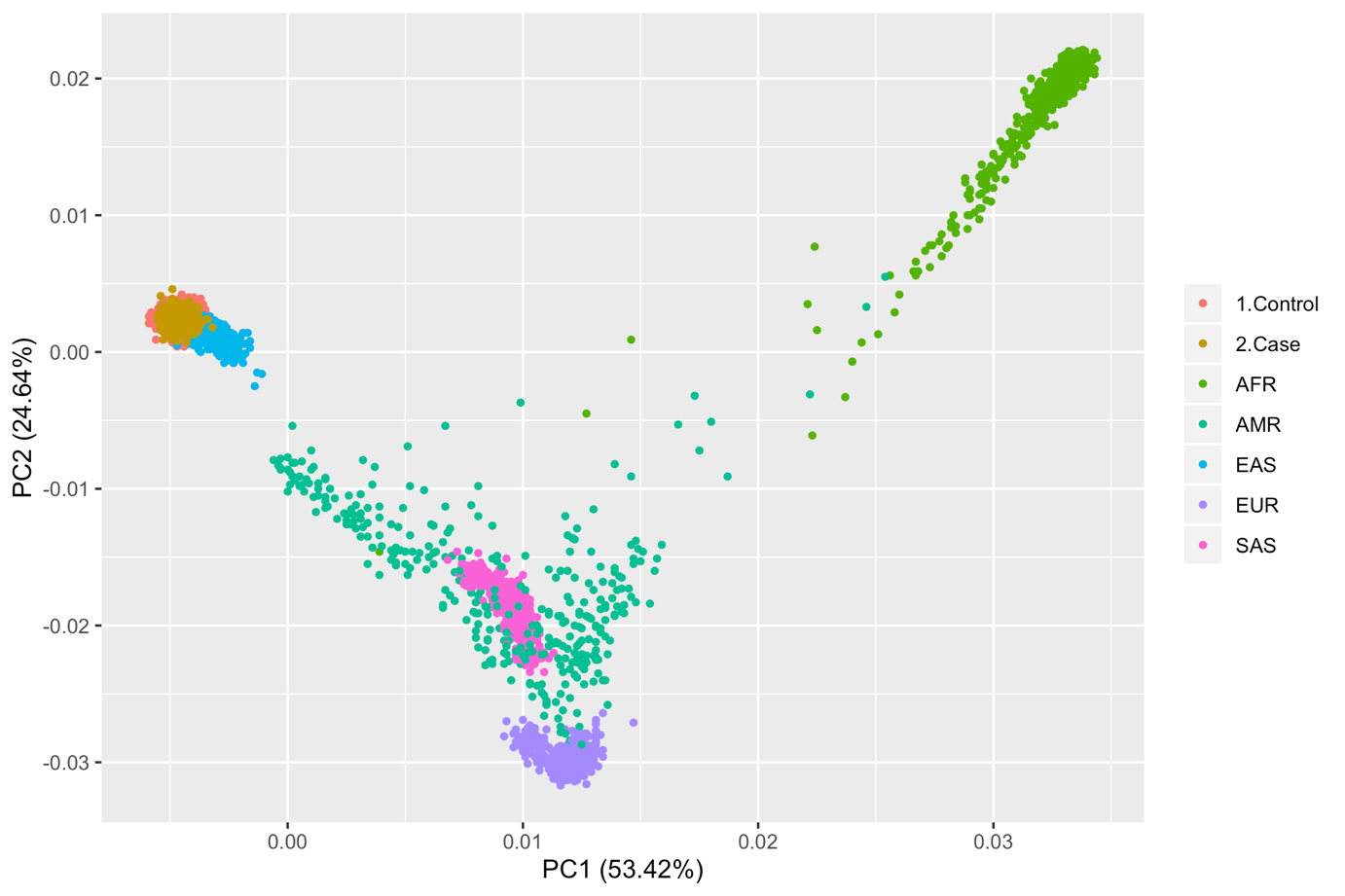


**Supplementary Figure 8.** Principle Component Analysis of case and control superimposed on 1k genome data. The case and control data cluster near EAS (East Asian) population.


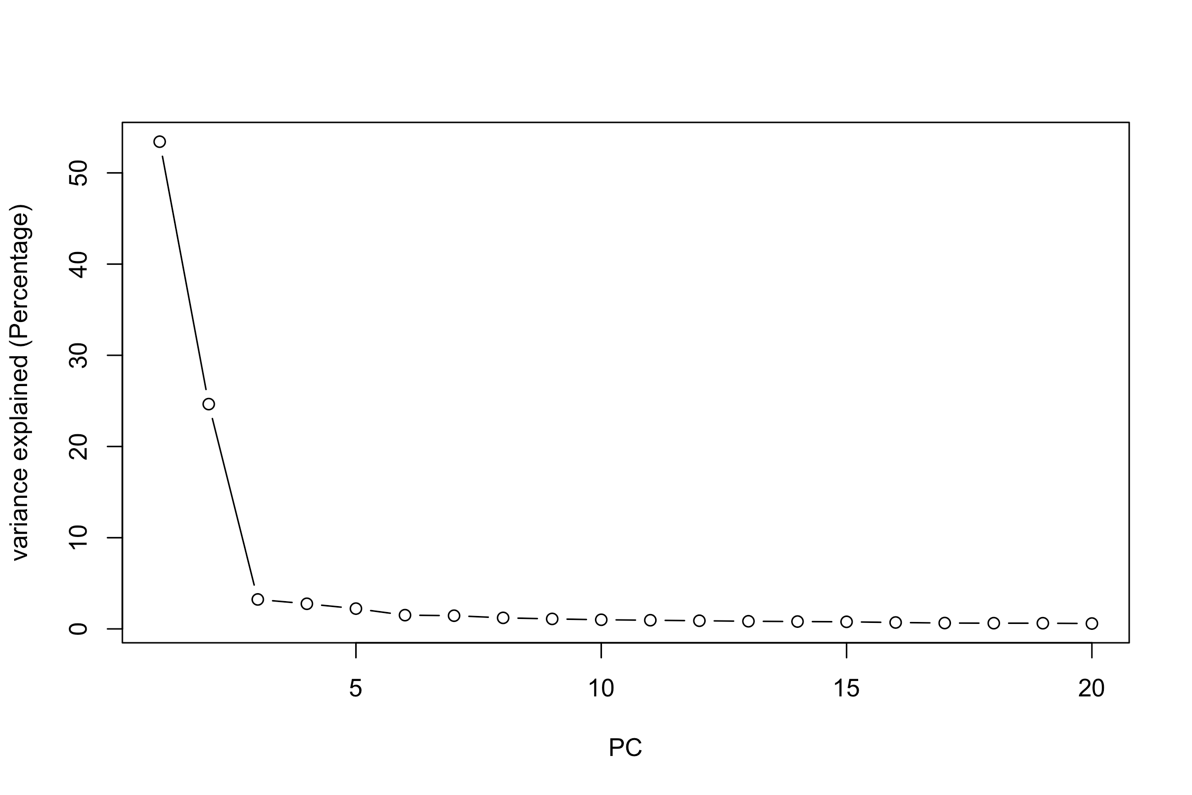


**Supplementary Figure 9.** Plot of top 20 Principal Components and variance explained by them.

**Supplementary Table 1.** 19 unique variants in 7 genes discovered to be significantly associated with prostate cancer.

| rsid | Gene | Build 38 position | ALT/REF | Amino Acid | MAF | MAF (case) | MAF (control) | Consequence | Impact | Polyphen | P_rm_ |
| --- | --- | --- | --- | --- | --- | --- | --- | --- | --- | --- | --- |
| rs148821008 | CDYL2 | 16:80633162 | T/C | V/I | 4.83E-04 (7) | 0 (0) | 5.59E-04 (7) | Missense | M | Benign (0.394) | 4.55E-07 |
| rs199772000 | CDYL2 | 16:80684586 | C/T | M/V | 6.88E-05 (1) | 5.04E-04 (1) | 0 (0) | Missense | M | Benign (0) | 0.690867 |
| rs200626206 | MST1R | 3:49902424 | A/G | R/* | 5.51E-04 (8) | 2.52E-03 (5) | 2.40E-04 (3) | Stop gained | H | - | 0.028018 |
| rs200757776 | MST1R | 3:49902631 | T/C | A/T | 2.41E-03 (35) | 3.02E-03 (6) | 2.31E-03 (29) | Missense | M | Possibly damaging (0.896) | 3.16E-05 |
| rs201008581 | MST1R | 3:49897281 | A/G | R/W | 2.74E-04 (4) | 5.04E-04 (1) | 2.39E-04 (3) | Missense,  Splice region | M | Benign (0.003) | 1.14E-05 |
| rs201024956 | MST1R | 3:49897557 | C/A | V/G | 1.20E-02 (173) | 1.16E-02 (22) | 1.20E-02 (151) | Missense | M | Probably damaging (0.936) | 1.21E-05 |
| rs202219889 | MST1R | 3:49887344 | T/C | R/Q | 1.86E-03 (27) | 3.02E-03 (6) | 1.67E-03 (21) | Missense | M | Benign (0.005) | 6.51E-05 |
| rs200476681 | ENTPD3-AS1 | 3:40411951 | T/G | M/I | 1.38E-04 (2) | 5.04E-04 (1) | 7.99E-05 (1) | Missense | M | Probably damaging (0.996) | 0.00012 |
| rs201157099 | ENTPD3-AS1 | 3:40415883 | A/C | T/N | 2.07E-04 (3) | 5.04E-04 (1) | 1.59E-04 (2) | Missense | M | Probably damaging (1) | 1.07E-05 |
| rs11544331 | GPER1 | 7:1091775 | T/C | P/L | 2.87E-02 (410) | 3.02E-02 (58) | 2.85E-02 (352) | Missense | M | Benign (0) | 1.31E-15 |
| rs181756759 | GPER1 | 7:1092777 | A/G | R/H | 2.07E-04 (3) | 1.51E-03 (3) | 0 (0) | Missense | M | Probably damaging (0.959) | 0.286629 |
| rs200626206 | LOC102724438 | 3:49902424 | A/G | R/* | 5.51E-04 (8) | 2.52E-03 (5) | 2.40E-04 (3) | Stop gained | H | - | 0.16888 |
| rs200757776 | LOC102724438 | 3:49902631 | T/C | A/T | 2.41E-03 (35) | 3.02E-03 (6) | 2.31E-03 (29) | Missense | M | Possibly damaging (0.896) | 3.1E-05 |
| rs200091077 | SPATA3 | 2:230996468 | T/C | R/C | 2.69E-03 (39) | 3.02E-03 (6) | 2.64E-03 (33) | Missense | M | Benign (0) | 1.97E-05 |
| rs202007096 | SPATA3 | 2:230996504 | A/G | V/M | 1.03E-03 (15) | 4.03E-03 (8) | 5.58E-04 (7) | Missense | M | Benign (0) | 0.552566 |
| rs145502673 | PARD3B | 2:204965221 | T/C | R/W | 3.43E-02 (494) | 3.32E-02 (65) | 3.45E-02 (429) | Missense | M | Probably damaging (0.958) | 3.99E-05 |
| rs150197749 | PARD3B | 2:205121680 | T/C | S/L | 1.45E-03 (21) | 1.00E-03 (2) | 1.52E-03 (19) | Missense | M | Benign (0.057) | 1.5E-05 |
| rs200533547 | PARD3B | 2:205499925 | A/G | R/H | 3.03E-03 (44) | 3.02E-03 (6) | 3.01E-03 (38) | Missense | M | Possibly damaging (0.84) | 5.3E-05 |
| rs201829385 | PARD3B | 2:205300662 | A/G | R/Q | 4.13E-04 (6) | 1.51E-03 (3) | 2.39E-04 (3) | Missense | M | Probably damaging (0.994) | 0.192618 |
| rs202115442 | PARD3B | 2:205158881 | G/A | I/V | 4.13E-04 (6) | 1.01E-03 (2) | 3.19E-04 (4) | Missense | M | Benign (0.003) | 0.000311 |
| rs2335704 | PARD3B | 2:205000109 | C/A | - | 1.20E-03 (29) | 2.52E-03 (5) | 1.91E-03 (24) | Intron variant | m | - | 1.3E-05 |

bbreviations: ALT: Alternate allele; REF: Reference allele; MAF: Minor allele frequency; M: Medium impact; H: High impact; m: Modifier; P_rm_: burden test p-value after removing variant from bin.

The table includes information on each variant - rsid, position, reference and alternate alleles, amino acid substitutions, MAF (minor allele frequency), MAF for cases and controls. The Consequence, Impact and Polyphen score are annotated using VEP. P_rm_ is the effective p-value when that variant is removed from the bin. The higher value of P_rm_ signifies higher contribution of the variant in the bin. The number enclosed by the brackets in MAF columns is the number of samples with rare variants in case, control and total study population.

In total, there were 2 high impact, 16 moderate impact and 1 modifier variant.

**Supplementary Table 2.** MAF of variants significantly associated with prostate cancer in Northeast Asians (Korea n =850, Mongolia n = 386, Japan, China, and Hong Kong) reference panel^25^.

| rsid | Build 38 position | Gene | MAF | MAF (NARD) | MAF (genomeAD) |
| --- | --- | --- | --- | --- | --- |
| rs148821008 | 16:80633162 | CDYL2 | 0.00048 | 0.00084 | 0.00009 |
| rs199772000 | 16:80684586 | CDYL2 | 0.00007 | - | 0.00014 |
| rs200626206 | 3:49902424 | MST1R | 0.00055 | - | 0.00002 |
| rs200757776 | 3:49902631 | MST1R | 0.00241 | 0.00197 | 0.00017 |
| rs201008581 | 3:49897281 | MST1R | 0.00028 | - | 0.00004 |
| rs201024956 | 3:49897557 | MST1R | 0.01198 | 0.01000 | 0.00041 |
| rs202219889 | 3:49887344 | MST1R | 0.00186 | 0.00112 | 0.00035 |
| rs200476681 | 3:40411951 | ENTPD3-AS1 | 0.00014 | 0.00112 | 0.00072 |
| rs201157099 | 3:40415883 | ENTPD3-AS1 | 0.00021 | 0.00112 | 0.00071 |
| rs11544331 | 7:1091775 | GPER1 | 0.02870 | 0.03300 | 0.20787 |
| rs181756759 | 7:1092777 | GPER1 | 0.00021 | - | 0.00010 |
| rs200626206 | 3:49902424 | LOC102724438 | 0.00055 | - | 0.00002 |
| rs200757776 | 3:49902631 | LOC102724438 | 0.00241 | 0.00197 | 0.00017 |
| rs200091077 | 2:230996468 | SPATA3 | 0.00269 | 0.00056 | 0.00045 |
| rs202007096 | 2:230996504 | SPATA3 | 0.00103 | - | 0.00011 |
| rs145502673 | 2:204965221 | PARD3B | 0.03434 | 0.04200 | 0.00200 |
| rs150197749 | 2:205121680 | PARD3B | 0.00145 | 0.00197 | 0.00017 |
| rs200533547 | 2:205499925 | PARD3B | 0.00303 | 0.00112 | 0.00011 |
| rs201829385 | 2:205300662 | PARD3B | 0.00041 | - | 0.00011 |
| rs202115442 | 2:205158881 | PARD3B | 0.00041 | - | 0.00016 |
| rs2335704 | 2:205000109 | PARD3B | 0.00200 | 0.01300 | 0.13500 |
